# The ameliorative potential of platelet-rich plasma and exosome on renal ischemia/reperfusion-induced uremic encephalopathy in rats

**DOI:** 10.1038/s41598-024-77094-2

**Published:** 2024-11-06

**Authors:** Hani M. Abdelsalam, Alaa Samy, Engy E. A. Mosaleem, Moustafa Salaheldin Abdelhamid

**Affiliations:** 1 Department of Zoology, Faculty of Science, Zagazig University, Zagazig, Sharkia 44519 Egypt; 2 Department of Surgery, Anesthesiology, and Radiology, Faculty of Veterinary Medicine, University of Mansoura, Mansoura, Egypt; 3 Department of Biochemistry, Faculty of Science, Zagazig University, Zagazig, Egypt

**Keywords:** Renal ischemia/reperfusion, Brain damage, Exosomes, Platelet-rich plasma, Uremic encephalopathy, In vivo, Biochemistry, Physiology

## Abstract

Uremic Encephalopathy results from the elevation of toxins and blood-brain barrier (BBB) disruption. Renal Ischemia/Reperfusion (I/R) injury is the principal cause of acute kidney injury and brain tissue injury. The present study was crafted to estimate the restorative impact of platelet-rich plasma (PRP) and exosome injection before the reperfusion phase on the kidney following renal I/R injury and its influence on brain tissue by tracking the histopathological, biochemical, and Doppler ultrasonography alternations in both kidney and brain tissue. Forty mature male rats were divided into five groups as follows: control, I/R, PRP, exosome, and Exosome + PRP. Renal Doppler ultrasonography was traced for all rats. Serum kidney functions and acetylcholine esterase enzyme (AchE) were evaluated. Both Gamma-aminobutyric acid (GABA) and glutamate were assessed in brain tissues. The oxidative stress (malondialdehyde), anti-oxidative (glutathione and catalase), and pro-inflammatory (Tumor necrosis factor- α and interleukin-6) markers were estimated in renal tissues. Additionally, morphometric histological examination was performed in both renal and brain tissues. Both PRP and exosome-received rats exhibited a significant improvement in both serum kidney functions and AchE compared to I/R rats. There was a 3.39-fold increase in GABA and a 2.27-fold decrease in glutamate levels in the brain tissue of PRP rats compared to the I/R rats. A significant elevation (*P* ≤ 0.0001) of glutathione and catalase besides a significant reduction in the expression of TNF-α and IL-6 was observed in renal tissue compared to I/R rats. A significant severe reduction (*P* < 0.0001) in the number of Purkinje cells, pyramidal cells in the cerebellar cortex, and the CA1 region in the hippocampus was observed in I/R rats which was significantly alleviated by both PRP and exosome. Furthermore, there was a significant improvement in Doppler parameters. PRP exerted a significant superior impact on the restoration of kidney functions and repairing uremic-induced damage in brain tissue.

**Supplementary Information:**

The online version contains supplementary material available at 10.1038/s41598-024-77094-2.

## Introduction

Renal I/R injury is a major risk factor that leads to acute kidney injury (AKI), which in turn is the eminent cause of rising rates of morbidity and mortality during major surgeries such as cardiac surgery, partial nephrectomy, kidney transplantation, vascular surgery, hemorrhagic shock, and other urological problems^1^. Renal I/R injury is defined as two conjugated complex stages: ischemia is a sudden cessation of blood flow, oxygen, and nutrients to kidney tissues, which stimulates the bioaccumulation of toxic nitrogenous wastes such as creatinine, urea, sodium, calcium, and a huge building up of reactive oxygen species (ROS) that is followed by parenchyma cell damage and likewise endothelial and tubular epithelial cells^2^. The renal ischemia phase is followed by the reperfusion phase, which is characterized by a huge influx of blood, re-oxygenation, and increasing P^H^ that adversely causes severe tissue damage and leads to inflammation, oxidative stress, impairment of energy metabolism, apoptosis, and tubular necrosis^3^.

The remote effect of renal I/R injury extends to other distant organs such as the pancreas, lungs, liver, and brain. Uremic encephalopathy results from uremia toxins elevation, an increase in serum sodium concentration that leads to a rising level of osmolality in brain tissue, activation of ROS expression, and suppression of the defense of antioxidant enzymes. All these successive events eventually result in BBB disruption, brain receptors, and neurotransmitter disarray^4,5^.

Platelet-rich plasma (PRP) is a pivotal autologous product extracted by centrifugation of a fresh blood sample to separate highly concentrated platelet-rich supernatant by an efficient, simple, and natural method^6^. Owing to the unique and double features of a huge number of effective growth factors included in PRP such as hepatocyte growth factor (HGF), insulin-like growth factor-1 (IGF-1), and epidermal growth factor (EGF) that carry multifactorial features in tissue regeneration, repair of renal tubules, proliferation of endothelial cells, curbing tubular necrosis, and renal healing after ischemia. Furthermore, neuroprotective effects of growth factors released from PRP express their impact by their ability to cross the blood-brain barrier^7^, for example, vascular endothelial growth factor (VEGF) that reported its role in blood-brain barrier permeability^8^, brain-derived neurotrophic factor (BDNF)^9^, nerve growth factor (NGF) via receptor-mediated transport^10^, and IGF-1^11^; So using PRP as a treatment tool in uremic encephalopathy is an important event.

Exosomes are a unique subtype of extracellular vesicles (ECVs), defined as heterogenous biovesicles with about 30–150 nanometers in diameter^12^. Exosomes are found in various biological fluids, such as urine, saliva, and breast milk. Almost all normal cell types can produce exosomes such as umbilical vein endothelial cells, T cells, Nature killer cells, and mesenchymal stem cells (MSC). Owing to its special cargo profile that is equipped with cellular signaling molecules such as lipids, proteins, and nucleic acid (mi RNA) and carries an anti-inflammatory, anti-apoptotic, and pro-angiogenic feature^13^, using MSC-derived exosomes is considered an attractive biological instrument in renal injury and neurodegenerative diseases compared to treatment with mesenchymal stem cells alone^14^. Consequently, this study aims to assess the potential ameliorative effect of PRP and exosome injection before the reperfusion phase on the kidney following renal ischemia or reperfusion injury and, subsequently, the distant influence on brain tissue.

## Material and method

### Ethical statement and sample size

All applications, treatments, and experimental procedures in the study were approved and adopted by the ethics of the Institutional Animal Care and Use Committee, Zagazig University, Zagazig, Egypt, with registration number ZU-IACUC/1/F/126/2023, which met the ARRIVE guidelines.

The achieved power of our study was calculated using G* Power (Germany) software version 3.1.9.4 using six experimental groups with a total sample size of 56 by post hoc power analysis depending on significant standards of numerator df = 5, denominator df = 50, α err prob = 0.05, and a critical F-value = 2.891 using ANOVA: one-way (one independent variable). This study’s effect size (f) was calculated from the results of all measurable tested variables, depending on the partial eta squared of each variance, and it ranged from 1.37 to 9.07. Results showed that the achieved power (1-β err prob) of this study equals 100%. Thus, the sample size of *N* = 56 is adequate to detect the hypothesis of the tested variables.

### Experimental design

**Fig. 1:**
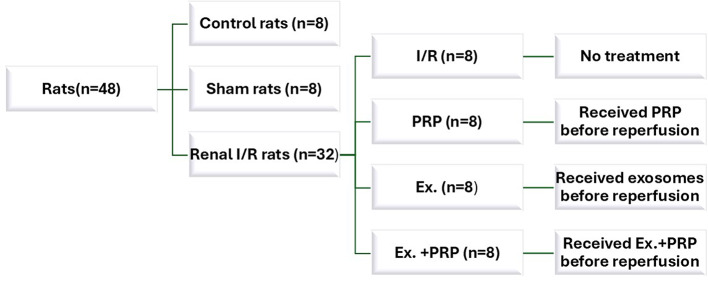
The study design of experimental groups; I/R, PRP, Ex., and Ex. +PRP groups.

A total of 56 mature male Sprague-Dawley rats, weighted (mean ± SD) 211.36 ± 5.32 g, were used in this controlled randomized experimental study. They were purchased and housed under standard conditions of a 12-h light/12-h dark cycle at 22 ± 2 °C and relative humidity of 65–70% at the Medical Experimental Research Centre (MERC), Faculty of Medicine, Mansoura University, Mansoura, Egypt. Animals were fed on a standard laboratory diet (from the department of nutrition, Faculty of Veterinary Medicine, Mansoura University, Mansoura, Egypt) with free access to water and food. A period of 7 days was allowed for rats to acclimatize to the new housing conditions. *Donor rats* (*n* = 8) were used for PRP preparation. *Experimental rats* (*n* = 48) were randomized into normal control rats (*n* = 8), sham rats (*n* = 8) that were subjected to laparotomy along with right nephrectomy without left renal clamping, and the whole rats exposed to I/R (*n* = 32) where left renal I/R injury along with right nephrectomy were performed, and later on, they were subdivided according to the received treatment into 4 groups (*n* = 8 each); I/R: received no treatment, PRP: received PRP before renal reperfusion, Ex.: rats received exosome before renal reperfusion, and Ex.+PRP: received PRP and exosome mixture before renal reperfusion Fig. 1.

### Preparation of exosome

Exosome® is a sterile, standardized concentration of lyophilized mesenchymal stem cell-derived exosome that were purchased from the Stem Vie® Company, Al Haram, Giza, Egypt. The exosome was diluted using the excipient Garamycin vial (2 ml), and thus, each 1 ml contains lyophilized concentrates of exosomes obtained from 3 × 106 mesenchymal stem cells and a protein concentration of BCA 500 µg/ml (+/- 10%).

### Platelet-Rich plasma (PRP) Preparation

Eight mature male Sprague-Dawley rats were used to prepare PRP, and platelets were counted according to the technique^15–17^. Briefly, fresh blood samples were collected under all aseptic conditions and anesthesia. To prevent clotting, samples were drawn into tubes containing 4% sodium citrate. The samples were then centrifuged using a laboratory centrifuge at 160 Xg for 20 min at an environmental temperature (22℃) to separate blood cellular elements, resulting in two layers: a lower red cell fraction and an upper straw-yellow turbid plasma fraction. The straw-yellow turbid supernatant layer was transferred to a clean tube and centrifuged once more at 400 Xg for 15 min, resulting in two components, two-thirds of the supernatant, platelet-poor plasma (PPP), that was discarded and the PRP retained. The number of platelets in PRP was counted to be 987,000 platelets/µl using CBC analyzer Sysmex XP300/Germany.

### Surgical procedures

All procedures were performed by the same trained and skilled surgeons (A.S. and E.A.) simultaneously at 10 AM to avoid the influence of both surgery variables and circadian rhythm on the results^18,19^.

**Fig. 2:**
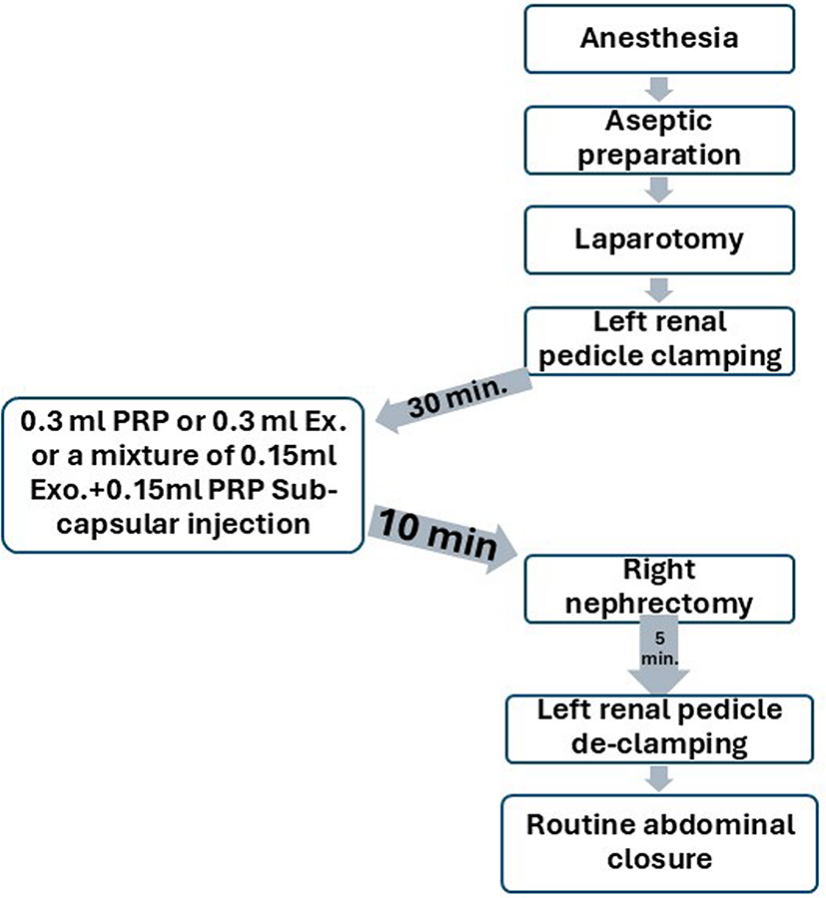
Step by step, the surgical procedures for induction and treatment of renal ischemia and reperfusion injury in all renal I/R groups (I/R, PRP, Ex., and Ex. +PRP groups).

**Fig. 3:**
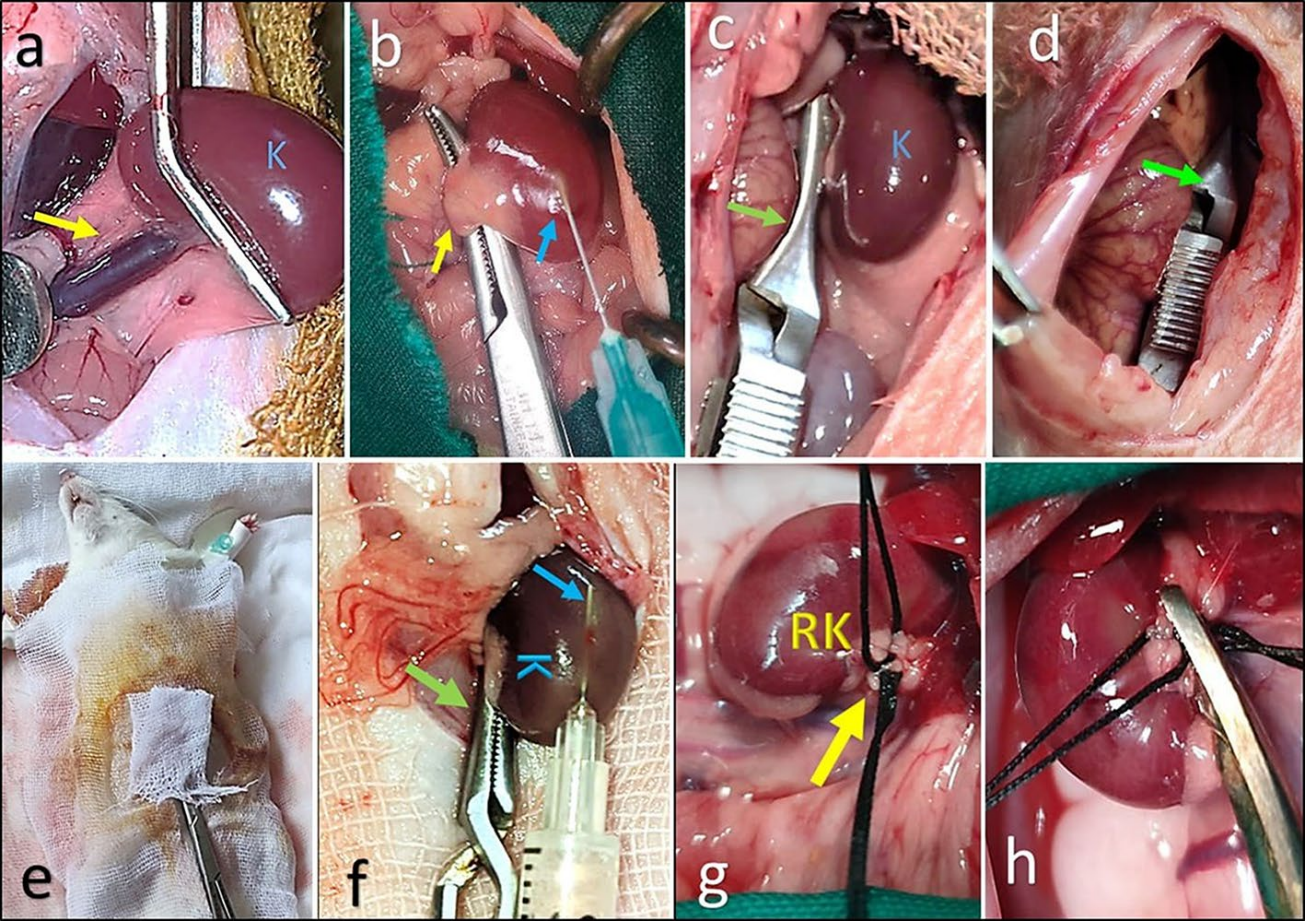
The surgical procedures used for the induction of renal ischemia/reperfusion injury. After laparotomy and approaching the left kidney (**a**), the renal pedicle (yellow arrow) was isolated (**b**) and clamped using a non-traumatic vascular bulldog (green arrow, **c**). Renal ischemia was maintained for 45 min by reinsertion of the clamped pedicle into its original situation (**d**) and covering the incisional line (**e**). Notice the careful subcapsular injection (blue arrow) before reperfusion in the congested kidney (**f**). A right nephrectomy was performed via double ligation of its pedicle and cutting in between (**g** and **h**). (blue K: left kidney, yellow RK: right kidney).

The surgical plane of general anesthesia was achieved via intraperitoneal injection of a mixture of 5 mg kg^-1^ Xylazine Hcl (20 mg/ml, XYLAJECT, ADWIA CO, Egypt) and 75 mg kg^-1^ Ketamine Hcl (50 mg/ml, KETAMINE, SIGMA TECH CO, Egypt)^20^. To induce left renal ischemia Figs. 2 and 3, the left renal pedicle was approached, isolated, clamped using a non-traumatic micro-vascular clamp, and reinserted in its position in the retroperitoneal cavity^1^. The abdominal skin incision was temporarily closed using small towel clamps to maintain ischemia for 45 min.

The left renal subcapsular injection with PRP and/or exosome was applied according to the treatment groups; PRP group (0.3 ml of PRP), Ex. group (0.3 ml of exosome), and Ex.+PRP group (0.15 ml PRP + 0.15 ml exosome) at 15 min before renal reperfusion. Through the same incision line, a right nephrectomy was performed 5 min before left renal reperfusion to prevent the right kidney from contributing to the results. Left renal reperfusion was achieved by careful de-clamping of the renal pedicle. Finally, the laparotomy incision was closed routinely by continuous patterns using 3/0 polyglycolide sutures (MAXON, COVIDIEN CO., USA), and the skin incision was closed with a 4/0 prolene suture Fig. 3. 

### Postoperative care

After the operation, rats were kept in an incubator at 37 °C for the next 6 h and then transferred into clean cages (2 rats per cage), with full access to food and water. Rats received an intramuscular injection of both the amoxicillin antibiotic (Flumox, EIPICO, Egypt) in a dose of 50 mg/kg and the anti-inflammatory meloxicam (Anti-cox II, 15 mg/3 ml, ADWIA, Egypt) in a dose of 5 mg/kg, two hours before the procedures and once a day afterward for the next three days. The wounds were dressed daily with povidone-iodine.

### Doppler ultrasonography imaging

Half an hour before euthanasia, each rat was prepared for ultrasound inspection by induction of general anesthesia using ketamine Hcl 75 mg kg^-1^ ip, shaving the entire abdominal area, and applying coupling gel. Non-invasive color Doppler ultrasound (Esaote MyLab 30X Vision, Esaote, Genova, Italy) was performed in all rats by the same person (A.S.) with a multi-frequency probe (6–12) with a filter of 100 Hz, pulse repetition frequency of 4,500 Hz, power of 50%, and a Doppler angle ranging from 0 to 40.

 Rats were examined and assessed transabdominal as previously mentioned^21^. Doppler signals of the left renal artery (before its division) were identified laterally close to the aorta and almost horizontally to the left kidney, posteriorly to the left renal vein, and at just the origin of the mesenteric cranial artery. The duration of each examination was about 5–10 min. The Doppler parameters: Resistance index (RI), Pulsatility index (PI), Peak systolic velocity (PSV), Time-average mean velocity (TAMV) and variables were determined and traced automatically. Two Doppler mages were traced and recoded from each rat.

### Collection of biological samples

After 3 days of reperfusion, rats were allowed to be placed in metabolic cages, and aseptically 24-hour urine samples were collected to evaluate the level of urinary parameters. Then all rats were sacrificed by a single overdose of thiopental sodium injected intra-peritoneally at 7.5 mg/100 g BW^22^. Using a sterile syringe, the whole blood of rats was obtained via cardiac puncture in a plain tube without anti-coagulant to allow the tubes to clot and centrifuged at 3000 rpm/min for 5 min. The upper serum was collected carefully and stored at -80 °C for further biochemical analysis. The left renal tissue was harvested and rinsed with cold saline to remove any clots or any red blood cells, then was cut into halves; one of the pieces was fixed in the buffered 10% formalin solution for histological procedures, and the other was stored at -80 °C for biochemical analysis. Briefly, renal tissues were prepared by washing with 1× PBS to remove excess blood, homogenized in 20 mL of 1× PBS, and then stored overnight at ≤ -20℃. After two freeze-thaw cycles, the homogenate was centrifuged at 5000×g for 5 min.

Brain tissue was removed, washed with cold saline, and then was cut into halves identically. The first portion was fixed in the buffered 10% formalin solution for histological examination, and the second one was frozen in liquid nitrogen and stored at -80 °C for further use. The tissue samples were homogenized after adding PBS (pH 7.4). Using centrifugation for 20 min at 2,000–3,000 rpm, the supernatant was collected carefully for the ELISA assay.

### Assessment of kidney function indicators

Serum levels of creatinine, uric acid (U.A.), and blood urea nitrogen (BUN) were analyzed using commercial kits (SPINREACT, Girona, Spain) by spectrophotometry and colorimetric assay at wavelengths of 492 nm, 340 nm, and 520 nm, respectively, according to the manufacturer’s protocol.

### Assessment of Acetylcholine esterase enzyme level (AchE)

AchE level was measured in serum using a rat-specific enzyme-linked immunosorbent assay, and the optical density was determined using a microplate reader at 450 nm (CUSABIO, Houston, U.S.).

### Determination of lipid peroxidation in renal tissue

The malondialdehyde level was measured using a commercial ELISA kit (MyBioSource, Vancouver, Canada) spectrophotometrically at wavelength 450 nm.

### Measurement of antioxidant biomarkers in renal tissue

Antioxidant parameters were measured using a commercial Rat Glutathione ELISA kit (CUSABIO, Houston, U.S.) and a commercial Rat Catalase ELISA kit (MyBioSource, Vancouver, Canada) spectrophotometrically at wavelength 450 nm.

### PCR analysis of TNF-α and IL-6 gene expression in renal tissue

The expression of IL-6 and TNF-α was measured using a real-time polymerase chain reaction (RT-PCR). The SYBR^®^ Green PCR Master Mix (Applied Biosystems, USA) was prepared according to the manufacturer’s instructions. Total RNA was isolated using a commercial kit (Promega, USA), Then mRNA was reverse transcribed into single-stranded cDNA using a cDNA synthesis kit (Meridian Bioscience, USA). Finally, the expression level of the relative mRNA was evaluated by calculating the values of $\Lambda$cycle threshold ($\Lambda$Ct) by normalizing the average values of Ct to the control housekeeping gene (GAPDH) and then calculating $2^{{ - \Lambda \Lambda Ct}}$ values.

### Measurement of neurotransmitters in brain tissue homogenate

A commercial ELISA kit (Bio Vision, Zurich, Switzerland) was used to estimate GABA concentration. Glutamate concentration was measured using a commercial ELISA kit (Abnova, Taiwan) at a wavelength of 450 nm using a microplate reader.

### Histological examination

After isolating the left kidney and brain from each rat, tissues were fixed in a 10% buffered formalin solution, paraffin-embedded, and sectioned into 3 μm-thick sections according to standard procedures. Gradually, the sections were deparaffinized, hydrated, and finally stained with hematoxylin and eosin (H&E). Then the samples were examined using a light microscope at ×400 magnification power by an experienced pathologist.

### Morphometry count

The morphometric change in left renal tissue was performed to evaluate the scoring of inflammation and congestion in the cortex and medulla of kidney tissue by counting degenerated tubules and casts formed after renal ischemia per 10 non-overlapping fields in each sample at ×400 magnification power using a light microscope by an experienced pathologist.

Correspondingly, the morphometric change in brain tissue was performed by counting the Purkinje cells in the cerebellar cortex and pyramidal cells in the CA1 region in the hippocampus per 10 non-overlapping fields in each sample at ×400 magnification power using a light microscope by an experienced pathologist.

### Statistical analysis

All data were expressed as the mean ± standard deviation (SD). The Statistical Package for the Social Sciences (SPSS) software, version 23.0 (Chicago, Illinois, USA), was used to perform all statistical analyses. The normality of the quantitative variables was determined using the Shapiro-Wilk test. A pairwise comparison of the tested quantitative variables between different groups was performed using a one-way analysis of variance (ANOVA), followed by Tukey’s post hoc correction. Whereas a difference with a chance probability of *P* < 0.05 was accepted as statistically significant. All graphs were carried out using GraphPad Prism software, version 8.0 (CA, USA).

## Results

### Doppler ultrasonography results

**Fig. 4:**
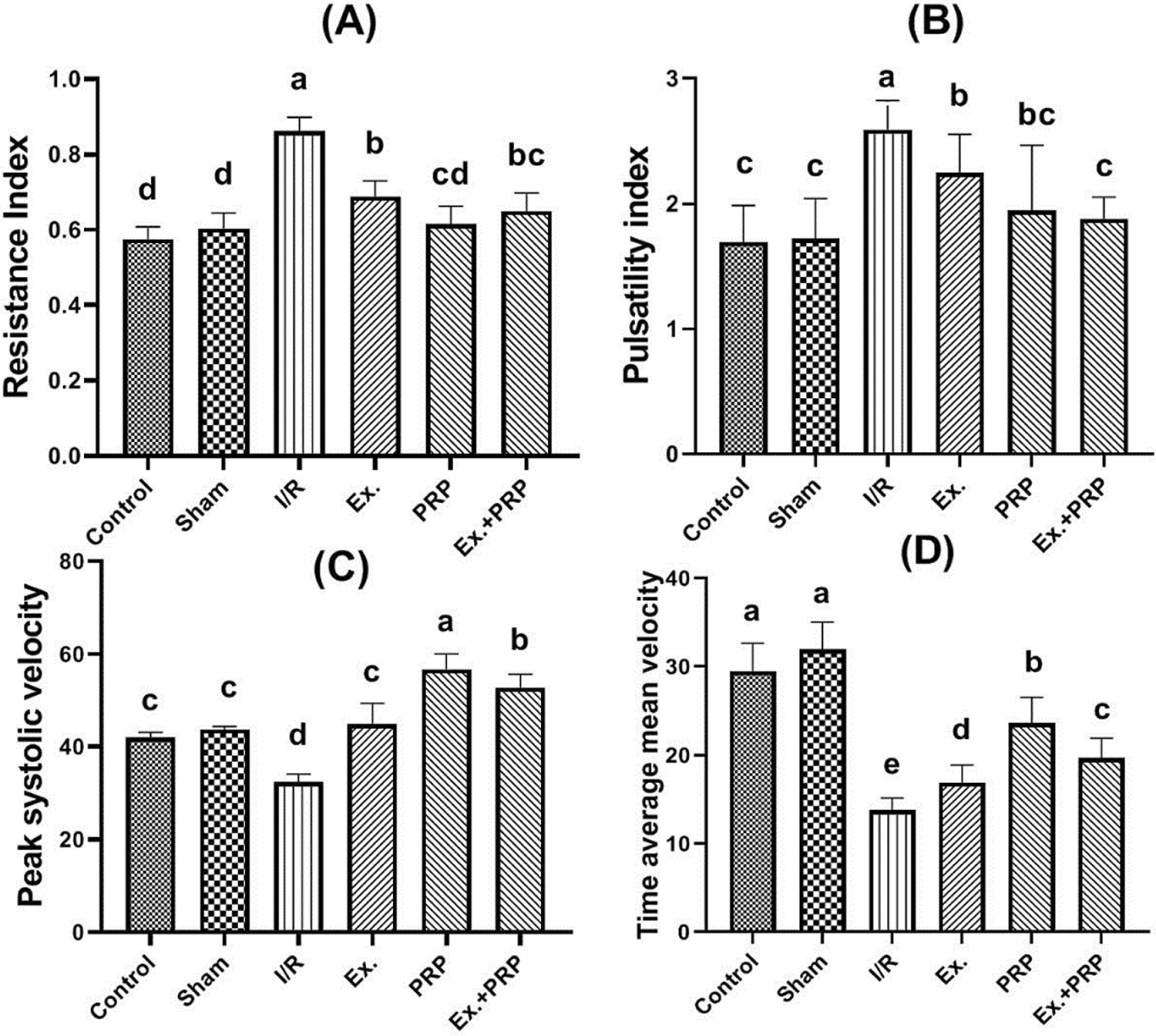
The effect of PRP and exosome administration on the level of RI (A), PI (B), PSV (C), and TAMV (D) of the renal artery. Statistical analysis using a one-way ANOVA followed by Tukey’s post hoc test. Variables were presented as the mean ± standard deviation (SD). Groups with different small superscript letters are significantly different at *P* < 0.05.

In the term of Doppler ultrasonography, results Fig. 4 showed a significant increase (*P* < 0.0001) in both renal RI and PI readings along with a significant decrease (*P* < 0.0001) in PSV and TAMV in I/R rats. Our finding showed that the entire treatment group exhibited significant (*P* < 0.0342) improvements in all Doppler parameters compared to those of the I/R group. PRP-treated rats revealed the most significant improvement in all Doppler parameters, and interestingly, both RI and PI of rats that received PRP were non-significant compared to those of the sham group (*P* = 0.9556, and 0.4069, respectively). Concerning both PSV and TAMV, compared to the sham group, results showed significant (*P* ≤ 0.0101) increases in their measurements in all treatment groups.

### Serum biochemical analysis

**Fig. 5:**
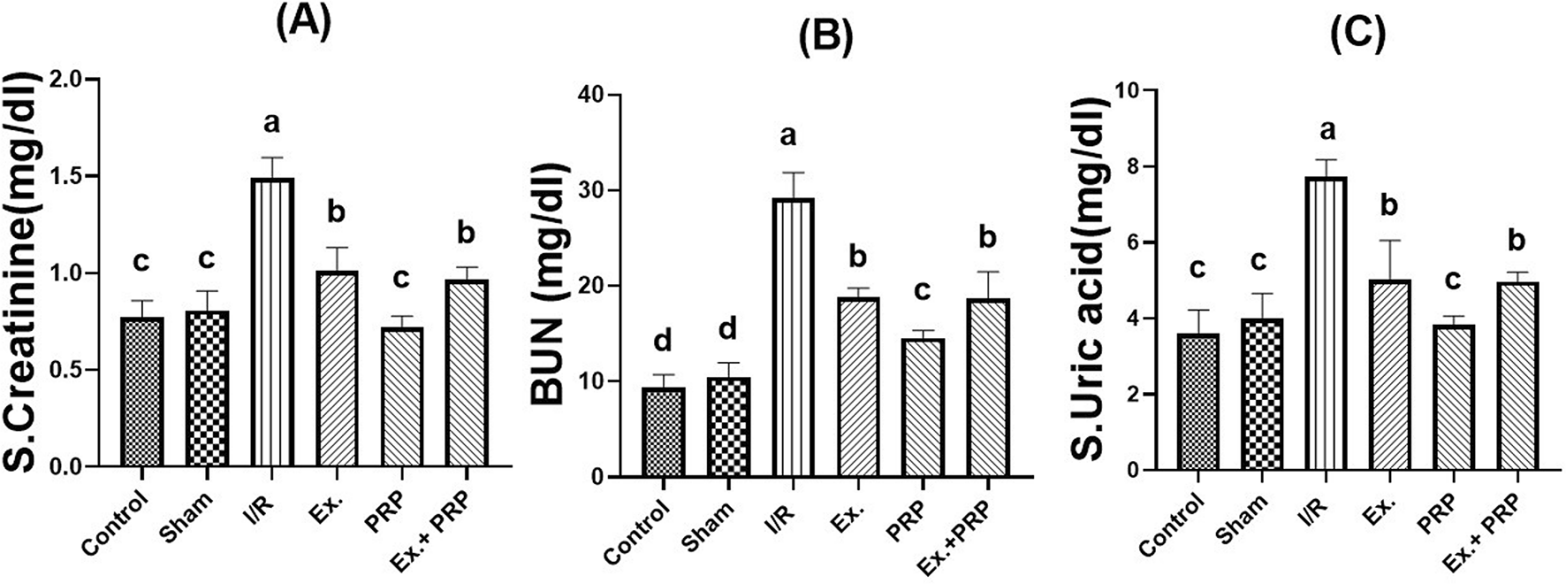
The effect of PRP and exosome administration on the levels of serum kidney function parameters. Statistical analysis using a one-way ANOVA followed by Tukey’s post-hoc test. Variables were presented as the mean ± standard deviation (SD). Groups with different small superscript letters are significantly different at *P* < 0.05.

As described in Fig. 5, compared with the sham group, creatinine, BUN, and uric acid serum levels were significantly (*P* < 0.0001) elevated in rats subjected to renal I/R injury. All treatment groups showed a significant (*P* < 0.0001) improvement in all tested serum kidney function parameters compared to the I/R group. PRP group revealed the most significant decrease (*P* < 0.0001) in serum creatinine, BUN, and uric acid than the other treatment groups, where it was non-significant (0.430-0.9942) from the sham group in the levels of creatinine and uric acid. Results revealed a non-significant effect between both Ex. and Ex.+PRP groups in enhancing serum creatinine, BUN, and uric acid.

**Fig. 6:**
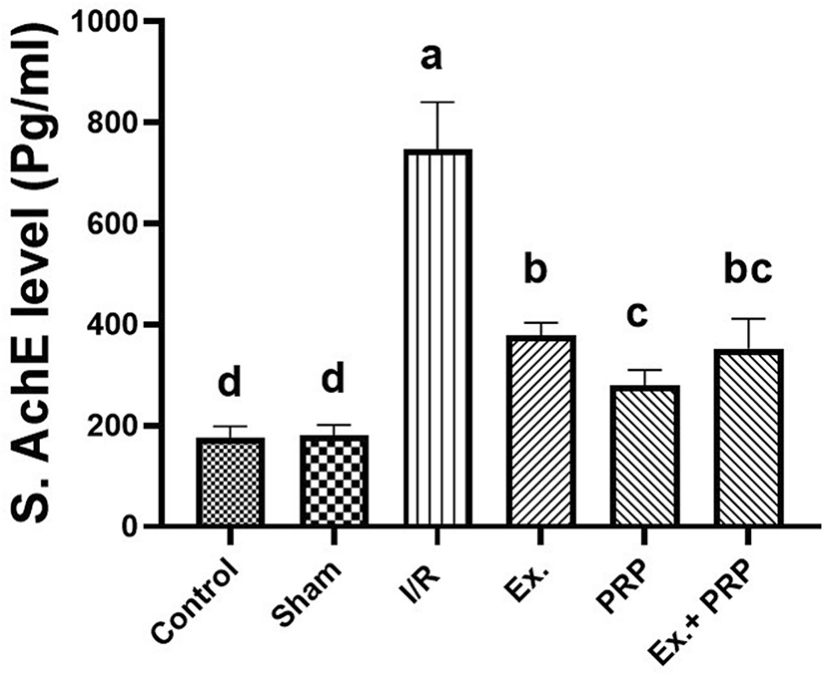
The effect of PRP and exosome administration on serum AchE level. Statistical analysis using a one-way ANOVA followed by Tukey’s post-hoc test. Variables were presented as the mean ± standard deviation (SD). Groups with different small superscript letters are significantly different at *P* < 0.05.

A significant (*P* < 0.0001) 4.2-fold elevation was recorded in rats of I/R group compared with the sham group. All treatment groups showed a significant (*P* < 0.0001) improvement in serum AchE measurement compared to the I/R group. Among the entire treatment groups, rats that received PRP were observed to have a significant (*P* < 0.0001) improvement in serum AchE level. Results revealed a non-significant effect between both Ex. and Ex.+PRP groups in enhancing serum AchE level Fig. 6.

### Results of oxidative and anti-oxidative statuses of renal tissue

**Fig. 7:**
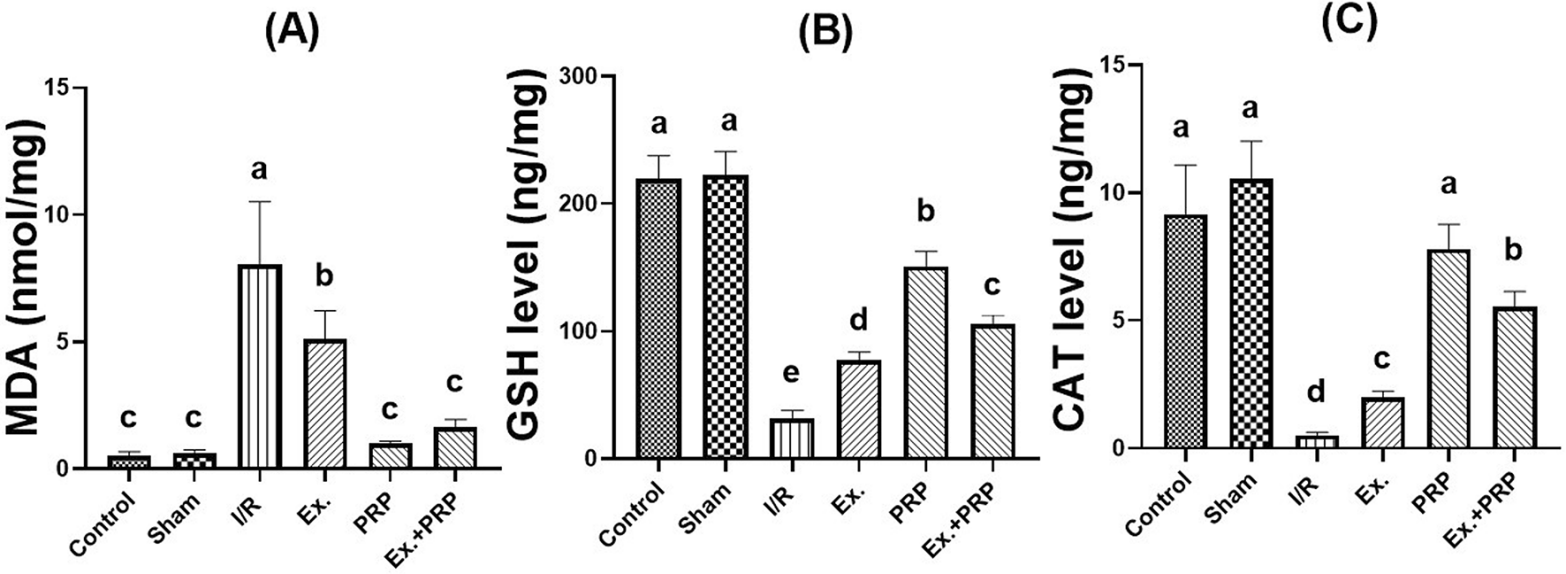
The effect of PRP and exosome administration on the levels of renal oxidative stress and antioxidant parameters. Statistical analysis using a one-way ANOVA followed by Tukey’s post-hoc test. Variables were presented as the mean ± standard deviation (SD). Groups with different small superscript letters are significantly different at *P* < 0.05.

As shown in Fig. 7, a significant (*P* < 0.0001) 12.85-fold increase in the oxidative stress marker MDA was observed in I/R rats compared with the sham rats. All treatment groups showed a significant (*P* < 0.0001) improvement in MDA measurement compared to the I/R group where both PRP and Ex.+PRP groups were non-significant (*P* = 0.4892–0.9841) from the sham group. Conversely, significant (*P* < 0.0001) 7.11- and 20.46-fold decreases were observed in GSH and CAT levels respectively in I/R groups compared to the sham rats. All treatment groups showed a significant (*P* < 0.0001) improvement in GSH and CAT measurement compared to the I/R group where PRP rats were non-significant (*P* = 0.0679) from the sham group in expression of CAT marker.

### Results of mRNA gene expression (IL-6 and TNF- α) in renal tissue

**Fig. 8:**
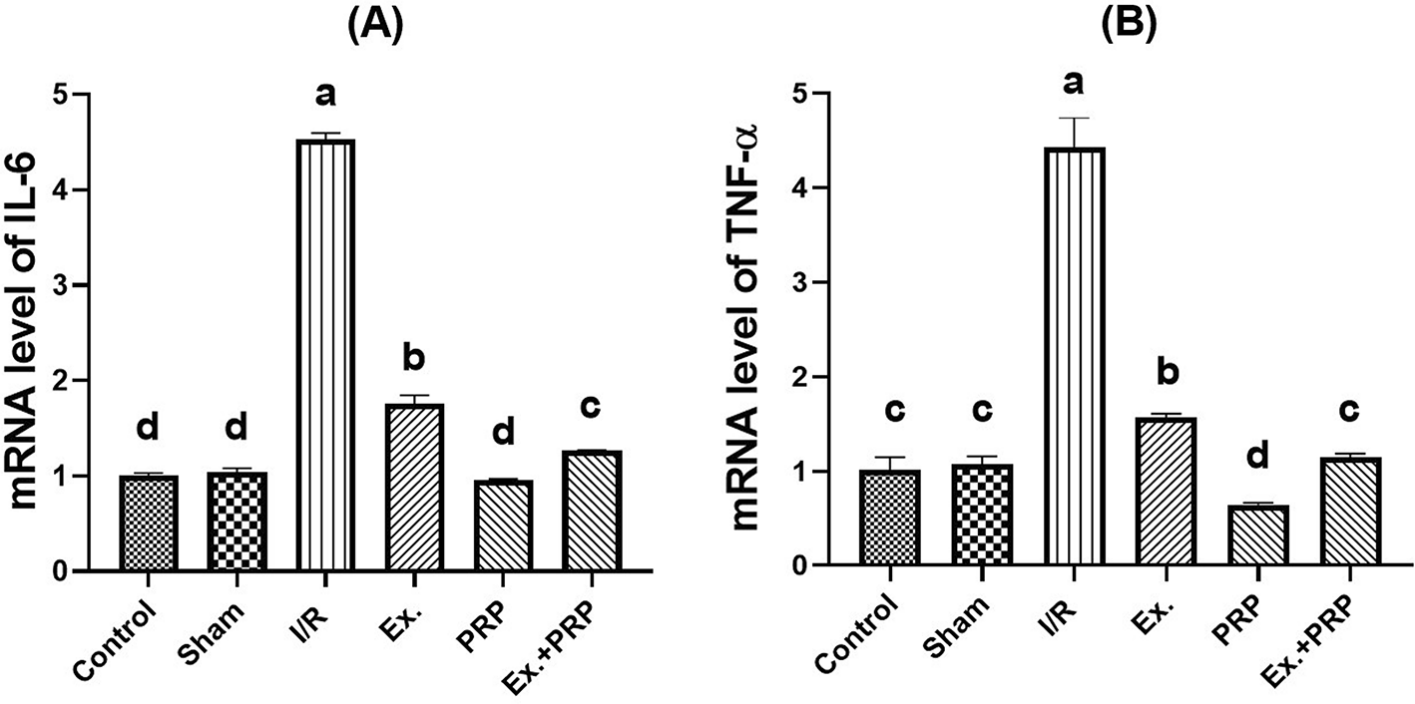
Real-time PCR analysis for IL-6 (**A**) and TNF- α (**B**) gene expression in kidney tissue. Statistical analysis using a one-way ANOVA followed by Tukey’s post hoc test. Variables were presented as the mean ± standard deviation (SD). Groups with different small superscript letters are significantly different at *P* < 0.05.

A significant (*P* < 0.0001) 4.35-fold, and 4.14fold increase in the mRNA expression levels of both pro-inflammatory mediators; IL-6, and TNF- α was observed in I/R rats compared with sham rats. All treatment groups showed a significant (*P* < 0.0001) improvement in IL-6, and TNF- α mRNA expression levels compared to the I/R group. PRP group revealed the most significant decrease (*P* < 0.0001) in IL-6 mRNA expression level than the other treatment groups, where it was non-significant (*P* = 0.0575) from the sham group as illustrated in Fig. 8.

### Results of neurotransmitter level (glutamate and GABA) in brain tissue

**Fig. 9:**
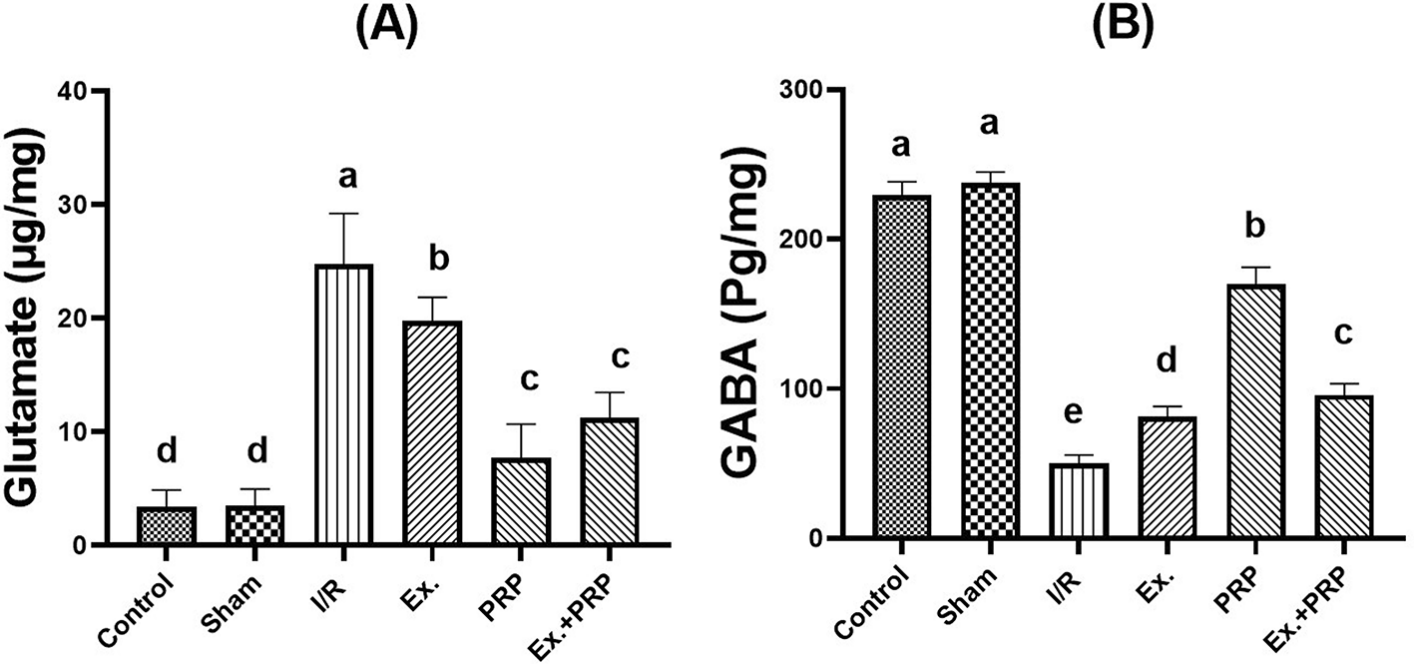
The levels of Glutamate (**A**) and GABA (**B**) neurotransmitters in the brain homogenate after administration of PRP and exosome. Statistical analysis using a one-way ANOVA followed by Tukey’s post-hoc test. Variables were presented as the mean ± standard deviation (SD). Groups with different small superscript letters are significantly different at *P* < 0.05.

As shown in Fig. 9, a significant (*P* < 0.0001) 7.028-fold increase in the level of glutamate was observed in I/R rats compared with the sham rats. All treatment groups showed a significant (*P* < 0.0001) improvement in glutamate level compared to the I/R group. Conversely, significant (*P* < 0.0001) 4.74-fold decreases were observed in GABA level respectively in I/R groups compared to the sham rats. All treatment groups showed a significant (*P* < 0.0001) improvement in glutamate and GABA measurement compared to the I/R group.

### Histopathological results

**Fig. 10:**
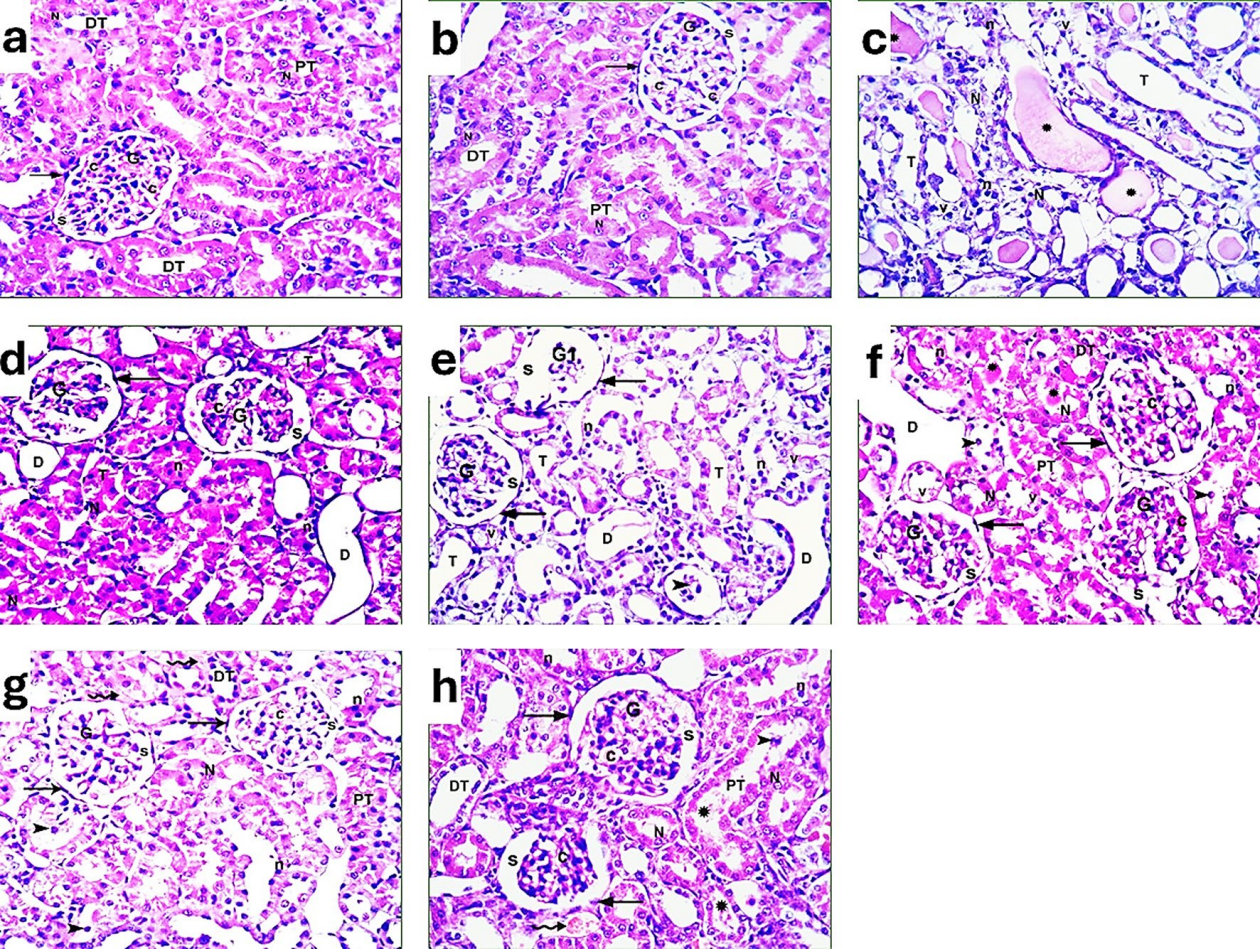
A photomicrograph of left renal cortex sections of adult male rats of different groups: (**a**): control group. (**b**): Sham group. (**c**, **d**, **e**): I/R group. (**f**): exosome group. (**g**): PRP group. (**h**): PRP + Ex. group (H&E, x400).

After examination of the renal cortex and medulla using light microscopy at (H&E, x400), it appeared that the renal cortex of a rat from the control group (distilled water and diet) shows a Malpighian corpuscle containing glomerulus (G) with many capillaries (c) and is surrounded by Bowman’s space (S), which is clear of debris. Bowman’s capsule reveals its parietal layer with flat squamous cells (arrow). Proximal (PT) and distal convoluted tubules (DT) appear with rounded vesicular nuclei, as explained in Fig. 10a. Regarding the sham-operated without inducing ischemia, the renal cortex of a rat shows a Malpighian corpuscle containing glomerulus (G) with many capillaries (c) and surrounded with Bowman’s space (S) which is clear of debris. Bowman’s capsule reveals its parietal layer with flat squamous cells (arrow). Proximal (PT) and distal convoluted tubule (DT) appear with rounded vesicular nuclei as shown in Fig. 10b. In the I/R group, the renal cortex of a rat shows enlarged Malpighian corpuscles with large, segmented glomeruli (G), congested capillaries (c), and is surrounded by a wide Bowman’s space (S) with debris. Bowman’s capsules reveal its parietal layer with flat cells (arrow). Nearly all tubules (T) appear with darkly stained nuclei (n), while a few tubules have rounded vesicular nuclei (N). Another corpuscle with atrophic glomeruli (G1) surrounded by a very wide Bowman’s space (S) is seen. Bowman’s capsules reveal its parietal layer with flat cells (arrow). Nearly all the tubules (T) appear with darkly stained nuclei (n) and vacuolated cytoplasm (v). Extensively dilated tubules (D) and numerous exfoliated cells in a tubule (arrowhead). Large acidophilic materials or casts (asterisks) fill their lumens, as observed in Fig. 10c, d, e.

Regarding the I/R rats injected with exosomes, the renal cortex shows a Malpighian corpuscle containing a glomerulus (G) with many capillaries (c) and is surrounded by a wide Bowman’s space (S) with debris. Bowman’s capsules reveal its parietal layer with flat cells (arrow). Proximal (PT) and distal convoluted tubule (DT) appear with rounded pale nuclei, while others have vacuolated cytoplasm (v), darkly stained nuclei (n), luminal exfoliated cells (arrowhead), or casts (asterisk), as demonstrated in Fig. 10f. The renal cortex section from the I/R rat injected with PRP reveals nearly normal tissue with little damage. Malpighian corpuscle containing glomerulus (G) with many capillaries (c) and surrounded with Bowman’s space (S) which is clear of debris. Bowman’s capsules reveal its parietal layer with flat squamous cells (arrow). Proximal (PT) and distal convoluted tubule (DT) appear with rounded nuclei (N) while others with darkly stained nuclei (n) and luminal exfoliated cells (arrowhead). Small capillaries (tailed arrows) are observed in Fig. 10g.

In I/R rats injected with PRP + Exosome, the renal cortex shows nearly normal tissue with little damage. Malpighian corpuscle containing glomerulus (G) with many capillaries (c) and surrounded by a wide Bowman’s space (S) with little debris. Bowman’s capsules reveal their parietal layer with cuboidal to flat cells (arrow). Proximal (PT) and distal convoluted tubules (DT) appear with rounded nuclei, while others have darkly stained nuclei (n), luminal exfoliated cells (arrowhead), or casts (asterisk). Wide congested capillaries (tailed arrows), as shown in Fig. 10h.

**Fig. 11:**
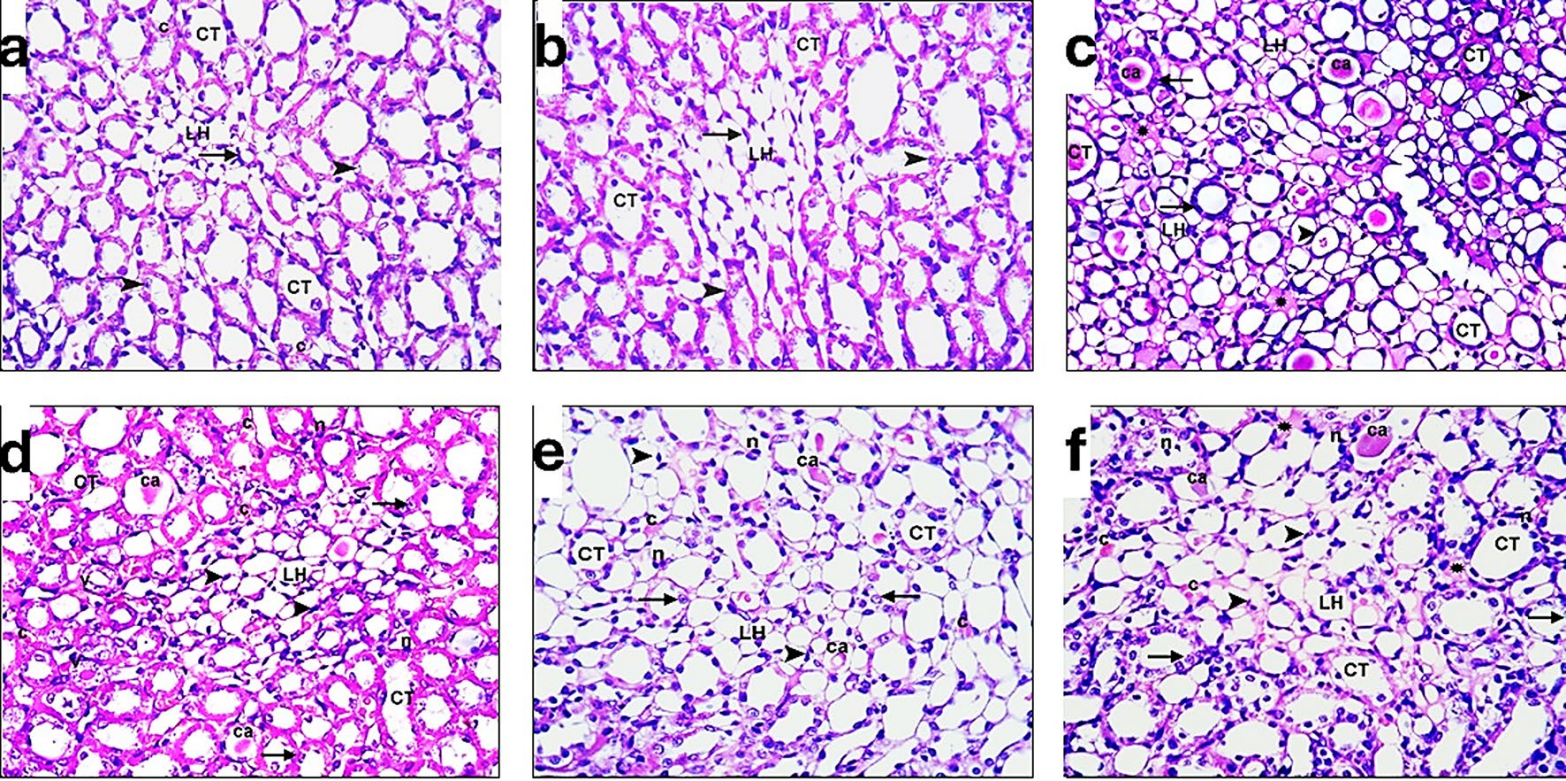
A photomicrograph of left renal medulla sections of adult male rats of different groups: (**a**): control group. (**b**): Sham group. (**c**): I/R group. (**d**): exosome group. (**e**): PRP group. (**f**): PRP + Ex. group (H&E, x400).

Concerning the renal medulla, rats in the control group (distilled water and diet) show a small group of loops of Henle (LH) with flat squamous lining cells (arrowhead). Most of the section shows collecting tubules or ducts (CT) with cuboidal lining cells (arrow), and a few small capillaries (c), as found in Fig. 11a. For the sham-operated without inducing ischemia rats, the renal medulla shows a small group of loops of Henle (LH) with flat squamous lining cells (arrowhead). Most of the section shows collecting tubules or ducts (CT) with cuboidal lining cells (arrow) as noticed in Fig. 10b. Rats in the I/R group (ischemia procedure) show a small group of loops of Henle (LH) with flat squamous lining cells (arrowhead). Most of the section shows collecting tubules or ducts (CT) with cuboidal lining cells (arrow). Nearly all tubules reveal darkly stained nuclei (n) and casts (ca.). Acidophilic or colloidal materials (asterisk) in between tubules and congested small capillaries (c), as noticed in Fig. 11c.

Regarding I/R rats-injected exosomes, it shows a group of loops of Henle (LH) with flat squamous lining cells (arrowhead). Most of the section shows collecting tubules or ducts (CT) with cuboidal lining cells (arrow). Numerous congested small capillaries (c), some tubules with vacuolated cytoplasm (v), and casts (ca.) are noticed in Fig. 11d. The renal medulla of the I/R rat-injected PRP shows a small group of loops of Henle (LH) with flat-lining cells (arrowhead). Most of the section shows collecting tubules or ducts (CT) with cuboidal lining cells (arrow). Few tubules with darkly stained nuclei (n) or casts (ca.) and small capillaries (c) are noticed in Fig. 11e.

In the I/R rats injected with PRP + Ex., the renal medulla shows a small group of Henle loops (LH) with flat squamous lining cells (arrowhead). Most of the section shows collecting tubules or ducts (CT) with cuboidal lining cells (arrow). Some tubules with darkly stained and exfoliated nuclei (n) or casts (ca.) and congested small capillaries (c) are noticed. Little acidophilic or colloidal materials (asterisks) in between tubules can be demonstrated in Fig. 11f.

**Fig. 12:**
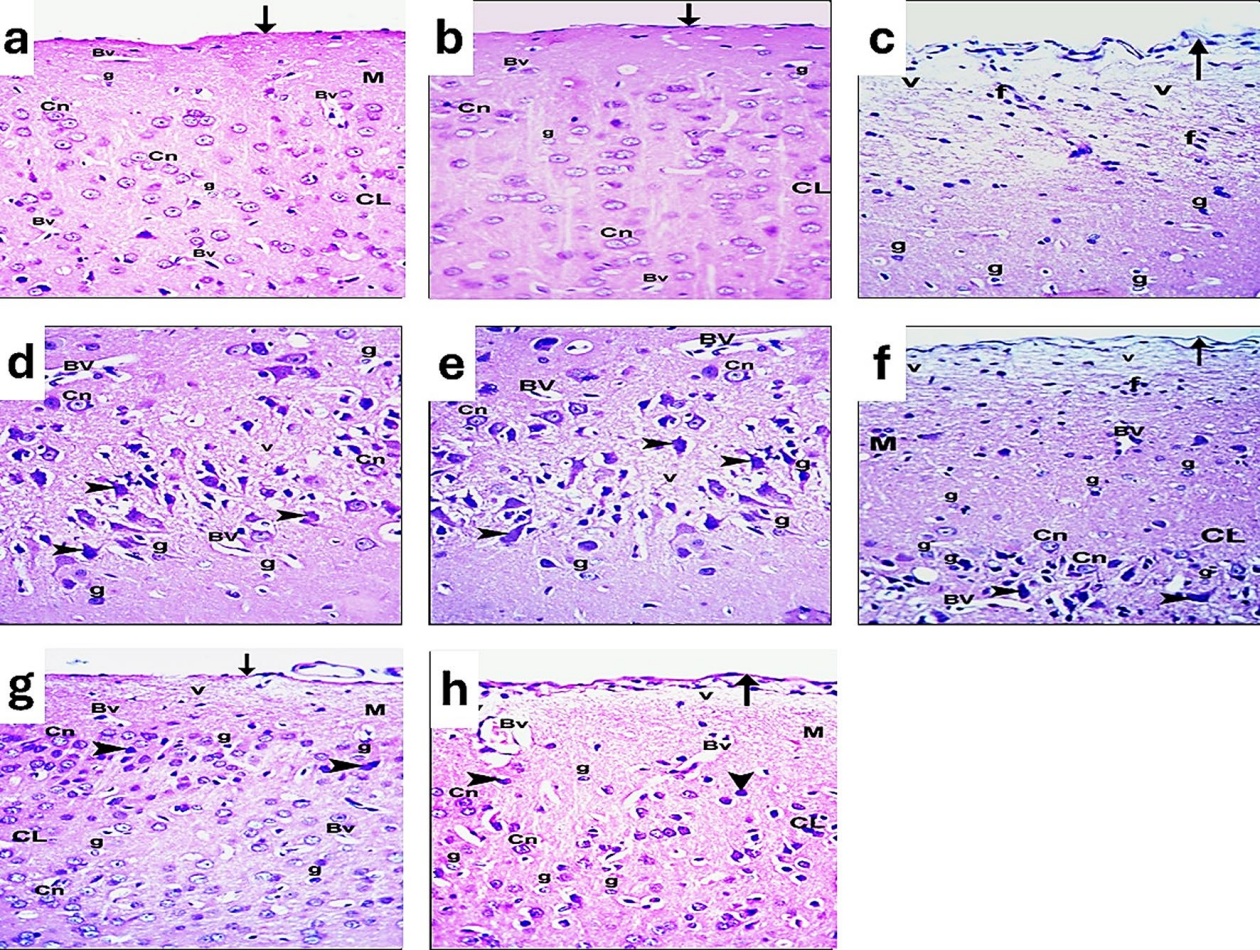
A photomicrograph of the cerebral cortex of brain sections of adult male rats of different groups: (**a**): control group. (**b**): Sham group. (**c**,** d**,** e**): I/R group. (**f**): exosome group. (**g**): PRP group. (**h**): PRP + Ex. group (H&E, x400).

On a light microscopic examination of the cerebral cortex at (H&E, × 400), the rat from the control group (distilled water and diet) shows closely adherent pia matter (arrow). It reveals the molecular layer and cellular external cortical layers (CL). Cortical neurons (Cn) with vesicular nuclei and basophilic cytoplasm are observed in these external layers. Intercellular neuropil shows neuroglia (g) and blood vessels with narrow perivascular spaces (Bv), as shown in Fig. 12a. Concerning the rats in the sham group, the cerebral cortex of the brain shows closely adherent pia matter (arrow). It reveals molecular layer cellular external cortical layers (CL). Cortical neurons (Cn) with vesicular nuclei and basophilic cytoplasm are observed in the external layers. Intercellular neuropil shows neuroglia (g) and blood vessels with narrow perivascular spaces (Bv) as observed in Fig. 12b. Regarding the rats in the I/R group, the cerebral cortex of a rat shows widely separated irregular pia matter (arrow). The outer thick molecular layer with different types of neuroglia (g), some inflammatory cells (f), and vacuolated neuropil (v), the external cortical layer. Cortical neurons (Cn) with vesicular nuclei and basophilic cytoplasm, numerous darkly stained shrunken neurons (arrowhead), and blood vessels with wide perivascular spaces (Bv), vacuolated neuropil (v), and different types of neuroglia (g) are observed in Fig. 12c, d, e.

In the Ex. group, the cerebral cortex of a rat shows separated pia matter (arrow) and the thick molecular layer (M) with some inflammatory cells (f) and a vacuolated neuropil (v). The external cortical layer (CL) shows cortical neurons (Cn) with vesicular nuclei and basophilic cytoplasm and numerous darkly stained shrunken neurons (arrowhead). Blood vessels with wide perivascular spaces (Bv) and different types of neuroglia (g) are observed in both the molecular and outer cortical layers, as illustrated in Fig. 12f. Regarding the I/R group of rats injected with PRP, the cerebral cortex of a rat shows nearly normal tissue. The closely adherent pia matter (arrow) is seen. Vacuolated neuropil (v) in the molecular layer (M) and cellular external cortical layers (CL). Most cortical neurons (Cn) appear with vesicular nuclei and basophilic cytoplasm, while a few darkly stained neurons (arrowheads) in the external layers are observed. Numerous different types of neuroglia (g) and blood vessels with narrow perivascular spaces (Bv) are found in Fig. 12g.

The cerebral cortex of I/R rats injected with PRP + Ex reveals more or less normal tissue. The slightly separated pia matter (arrow) is seen. The outer molecular layer (M) shows vacuolated neuropil (v) and blood vessels with wide perivascular spaces (Bv). The external cortical layers show most cortical neurons (Cn) with vesicular nuclei and basophilic cytoplasm and a few darkly stained neurons (arrowhead). Different types of neuroglia (g) are observed in this all-layers, as demonstrated in Fig. 12h.

**Fig. 13:**
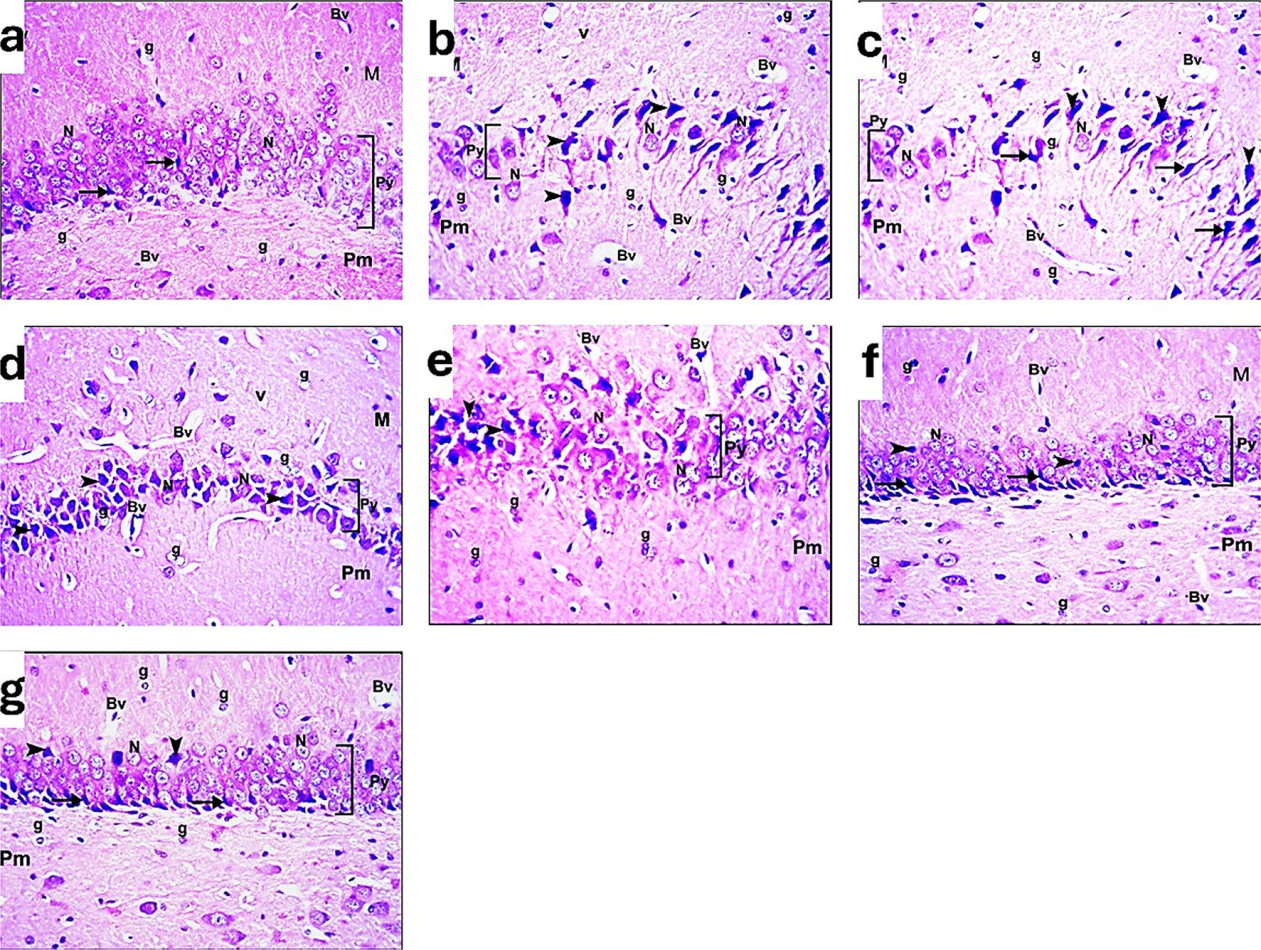
A photomicrograph of the CA1 region of the hippocampus of brain sections of adult male rats of different groups: (**a**): control group. (**b**): sham group. (**c**, **d**): I/R group. (**e**): exosome group. (**f**): PRP group. (**g**): PRP + Ex. group (H&E, x400).

Regarding the control group of rats (distilled water and diet), the CA1 region of the hippocampus reveals both molecular (M) and polymorphic (Pm) layers, with some glial cells with pale nuclei (g) and blood vessels with narrow perivascular spaces (Bv). 5–6 compact layers of pyramidal neurons with centrally vesicular nuclei (N) and a few glial cells with elongated, darkly stained nuclei (arrow) are observed in the pyramidal layer (Py) in Fig. 13a. For sham-operated rats, the CA1 region of the hippocampus shows polymorphic (Pm) layers having some glial cells with pale nuclei (g) and blood vessels with narrow perivascular spaces (Bv). 5–6 compact layers of pyramidal neurons with centrally vesicular nuclei (N) and a few glial cells with darkly stained nuclei (arrow) are observed in the pyramidal layer (Py) as shown in Fig. 13b. In the I/R rats, the CA1 region of the hippocampus is shown in the pyramidal layer (Py); 2–3 layers of few normal neurons with centrally vesicular nuclei (N), others shrunken with darkly stained nuclei (arrowhead), and numerous glial cells with elongated darkly stained nuclei (arrow). Both molecular (M) and polymorphic (Pm) layers show numerous different types of glial cells (g) and also blood vessels with wide perivascular spaces (Bv), as noticed in Fig. 13c, d.

The CA1 region of the hippocampus from the group of I/R rats injected with exosomes shows a few hypocellular layers of pyramidal neurons with centrally vesicular nuclei (N) and numerous other neurons with darkly stained nuclei (arrowheads) in the pyramidal layer (Py). Both molecular (M) and polymorphic (Pm) layers show numerous glial cells with pale nuclei (g) and blood vessels with wide perivascular spaces (Bv), as shown in Fig. 13e. Moreover, in I/R rats injected with PRP, the CA1 region of the hippocampus shows nearly normal tissue. 3–5 layers of pyramidal neurons with centrally vesicular nuclei (N) and numerous glial cells with elongated, darkly stained nuclei (arrow) are observed in the pyramidal layer (Py). Both molecular (M) and polymorphic (Pm) layers show numerous glial cells with pale nuclei (g) and blood vessels with narrow perivascular spaces (Bv), as observed in Fig. 13f.

In PRP + Ex. injected group of rats, the CA1 region of the hippocampus shows nearly normal tissue. 3–5 layers of pyramidal neurons with centrally vesicular nuclei (N) and numerous glial cells with elongated, darkly stained nuclei (arrow) are observed in the pyramidal layer (Py). Both molecular (M) and polymorphic (Pm) layers show numerous glial cells with pale nuclei (g) and also blood vessels with wide perivascular spaces (Bv), as stated in Fig. 13g.

### Morphometry results

**Fig. 14:**
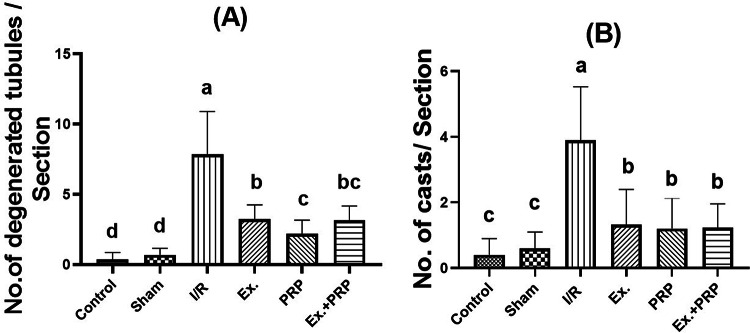
The effect of PRP and exosome administration on the change of the number of degenerated tubules and casts in the renal cortex. Statistical analysis using a one-way ANOVA followed by Tukey’s post hoc test. Variables were presented as the mean ± standard deviation (SD). Groups with different small superscript letters are significantly different at *P* < 0.05.

**Fig. 15:**
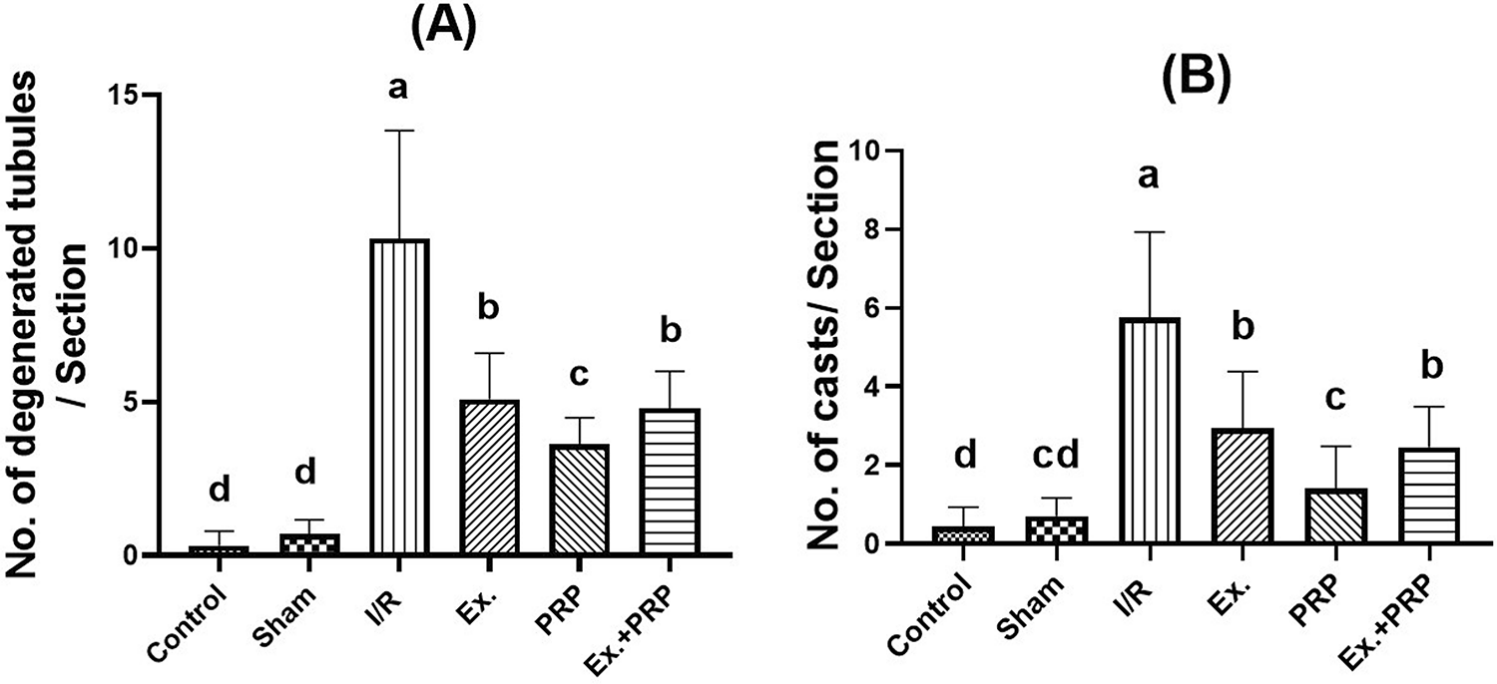
The effect of PRP and exosome administration on the change of the number of degenerated tubules and casts in the renal medulla. Statistical analysis using a one-way ANOVA followed by Tukey’s post hoc test. Variables were presented as the mean ± standard deviation (SD). Groups with different small superscript letters are significantly different at *P* < 0.05.

After examination and counting 10 non-overlapping fields at 400X magnification power using a light microscope by an experienced pathologist, it was recorded that the number of renal degenerated tubules in both cortex and medulla was increased compared to the sham rats, owing to the fact that the rats in renal I/R groups were subjected to 45 min ischemia that ultimately leads to congestion and inflammation of renal tissue. Concerning the formation of casts, a high increase in their number after renal I/R injury was recorded. The formation occurred due to disruption in renal tubules, then enlargement in Malpighian corpuscles and a wide Boman’s capsule, which in the end led to a low filtration rate and the accumulation of toxins and casts Figs. 14 and 15.

**Fig. 16:**
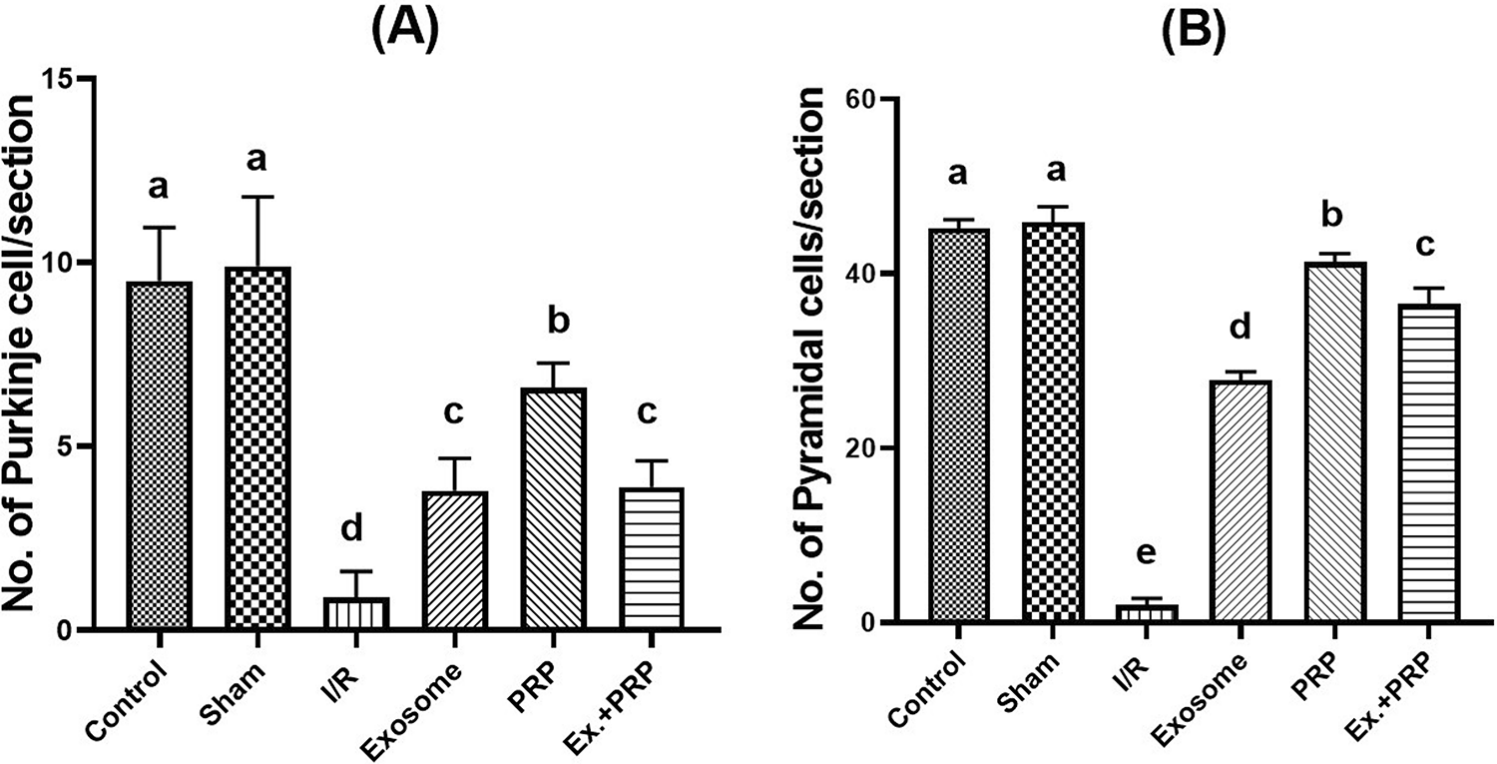
The effect of PRP and exosome administration on the change of the number of Purkinje cells in the cerebellar cortex and pyramidal cells in the CA1 region in the hippocampus of brain tissue. Statistical analysis using a one-way ANOVA followed by Tukey’s post hoc test. Variables were presented as the mean ± standard deviation (SD). Groups with different small superscript letters are significantly different at *P* < 0.05.

As illustrated in Fig. 16, after examination and counting 10 non-overlapping fields at 400X magnification power using a light microscope by an experienced pathologist, it was recorded that the number of Purkinje cells, pyramidal cells in the cerebellar cortex, and the CA1 region in the hippocampus, respectively, was highly reduced compared to the sham group (*P* < 0.0001).

## Discussion

The main target of the present study was to investigate the protective effects of renal sub-capsular injection of PRP and MSC-derived exosome for the first time especially at the pre-reperfusion phase on the remote impact of brain functions following a renal I/R injury. Treatment with PRP in this model was associated with a superior improvement in both renal and distant effects on brain biomarkers and functions. In contrast, rats treated with exosomes show the significantly weakest effect among the treatment groups in both kidney and brain functions. The combination group exhibited a significant improvement in the bio-physiological markers of both the kidney and brain compared to the sham one, but this improvement was significantly weaker than the PRP group.

Nephrotoxicity not limited only by induction renal I/R model but also there are various potent medicals that had various side effects such as Methotrexate^23^, and Cisplatin^6,24^, the later one mentioned has been reported from previous studies its role in nephrotoxicity after accumulation in renal proximal tubules that converted to other toxic reactive metabolite, a cysteine conjugate, that act as a terrible promoter for cellular kidney injury. MSCs had reported its effect as immunomodulatory, anti-oxidant, anti-inflammatory tool in injured kidney tissue whether injury induced by cisplatin^24^ or establishment a renal I/R injury model^13^.

Renal ischemia/reperfusion triggered a complicated sequence of events initiated in the ischemic phase by deprivation of the tissue from nutrients and oxygen supply, then switching off the aerobic cellular metabolism that turns into anaerobic conditions, consequently reducing the rate of ATP production, Na^+^/K^+^ ATPase dysfunction, and ATPase pumping that leads to Na^+^ accumulation in the cytoplasm and ultimately hypertension^25^. In the present study, our kidney function results revealed that induction renal I/R markedly increased levels of serum creatinine, BUN, and uric acid. Thus, consistent with previous studies mentioned above, our findings have proved that rats in both groups were injected with PRP and Ex. +PRP before the reperfusion phase revealed more improvement in all biochemical parameters and histological tissues owing to the unique features of PRP than exosomes alone.

Whereas rats injected with PRP before reperfusion exhibited a superior significant enhancement in serum kidney function parameters by decreasing levels of serum creatinine, BUN, and uric acid, and ameliorates hypertension, compared with I/R rats. Gradually, the improvement was recorded but by a less significant degree in Ex. +PRP rats than others in the PRP treated rats before reperfusion.

The levels of serum kidney function parameters, creatinine, BUN, and uric acid, were reduced. As the concerned group of rats was injected with exosomes only, and owing to the rapid clearance, low retention, and low encapsulation capacity features of exosomes that impede the effect of injected exosomes^26,27^. The rats received a single dose of exosomes before reperfusion, which revealed the weakest effect on both kidney function parameters.

Furthermore, during the reperfusion phase, the aerobic metabolism resumed with rapid elevation in O_2_ and P^H^ normalization, Ca^++^ overload that causes damage in the mitochondrial membrane, and overproduction of reactive oxygen species (ROS) that causes an imbalance between the antioxidant defense system by decreasing GSH content and catalase enzyme levels and increasing oxidative cellular stress, including high levels of MDA as a final by-product of lipid peroxidation during β-oxidation of unsaturated fatty acids^28^. Alongside previous studies, it was recorded that in renal homogenate of I/R group, enhancement of lipid peroxidation by elevation level of MDA as well as decreasing the level of both antioxidants CAT enzyme and GSH. Our results proved that rats in both groups were injected with PRP, and Ex. +PRP before the reperfusion phase revealed more improvement in all antioxidant and oxidative stress parameters. Correspondingly, a great elevation was noticed in the antioxidant defense system by increasing both levels of CAT enzyme and GSH.

Lipid peroxidation in renal tissue was significantly reduced by decreasing the level of MDA in the renal tissue of PRP-treated rats compared with the I/R rats group. Progressively, the improvement was reduced in the group of rats injected with a mixture of platelet-rich plasma and exosomes before reperfusion compared to others in the group of rats injected with PRP before reperfusion; the level of CAT antioxidant enzyme and GSH level significantly increased after injection rats with PRP + Ex. before reperfusion, on the other hand, the oxidative stress was ameliorated by decreasing the level of MDA. Regarding the I/R rats injected with exosomes, only the weakest effect was shown owing to the limitations mentioned above based on previous studies; CAT antioxidant enzyme and GSH level increased significantly after injection with exosomes before reperfusion compared to I/R rats. Additionally, the level of MDA as a biomarker for lipid peroxidation increased significantly.

Owing to the successive damage induced by the period of ischemia in renal I/R tissue, the inflammatory reactions begin through the high expression of proinflammatory cytokines TNF-α and IL-6 in I/R renal tissue^29^. In line with these studies, our findings were recorded that in renal homogenate, there was a markedly increased mRNA expression level of the anti-inflammatory cytokines TNF-α and IL-6 by promoting oxidative stress and a high expression of adhesion molecules in renal tissue^30,31^. By virtue of the superior efficiency of injection PRP before reperfusion, our results proved that renal I/R injected with PRP had highly superior effects by reducing the elevated mRNA expression level of pro-inflammatory cytokines TNF-α and IL-6 compared with the I/R group. For the group of rats injected with Ex. +PRP, the elevated mRNA expression level of pro-inflammatory cytokines TNF-α and IL-6 was reduced, but to a lesser degree than in rats injected with a single dose of PRP. Relative to a Ex. -treated rats before reperfusion, the levels of proinflammatory mediators TNF-α and IL-6 were significantly reduced by the weakest degree among other groups.

At the same time, renal histological deterioration was exhibited by the enlargement of the Malpighian corpuscles with large, segmented glomeruli, congested capillaries, and a wide Bowman’s space with a reduction glomerular filtration rate, leading to the accumulation of uremic toxins and damage to renal proximal TECs because of their high demand for O_2_ supply in the cortex and medulla^32,33^. In our study, it was proven that renal histological deteriorations were enhanced and restored to normal rats in the control group after renal subcapsular injection with PRP, owing to its superior regeneration power for its growth factors. Moreover, the morphometry examination was a vital confirmatory tool in counting the number of degenerated tubules and casts in both the renal cortex and medulla after their extremely elevating level in I/R rats.

Our findings explained that in rats subjected to renal I/R without treatment, distant inflammatory damage to brain tissue was evidenced by increasing levels of serum acetylcholine esterase enzyme, neurotransmitter derangement by increasing levels of glutamate, and decreasing levels of GABA in brain homogenate. Regarding the PRP-treated rats injected with before reperfusion, an enhancement in brain biochemical parameters was observed by a significant reduction in the hyperactivity of the serum acetylcholine esterase enzyme, the level of glutamate in brain homogenate, and the increasing level of GABA in brain tissue compared to I/R rats.

Subsequently, the amelioration observed in rats injected with Ex. +PRP to a low degree in the brain was achieved by decreasing the increased levels of both serum acetylcholine esterase and glutamate levels in brain tissue and increasing the level of GABA in brain homogenate compared with the I/R group. Due to the limitations faced by exosome injection, the weakest effect was recorded in the rats injected with a single dose of exosome before reperfusion: a significant decrease in serum acetylcholine esterase level, glutamate in brain homogenate, and a noticeable increase in GABA level.

The distant effect of renal I/R injury extends to other organs such as the pancreas^34^, lungs^35^, liver^36^, and brain^5,37^ have been reported in various previous studies. In harmony with these findings, our results were interpreted as follow by elevating levels of uremic nitrogenous wastes, including guanidino compounds, creatinine, urea, and uric acid, as well as an increasing level of serum Na^+^, oxidative stress, and inflammatory mediators with suppression of anti-oxidant defense enzymes. All these factors contribute to brain tissue inflammation and neuronal loss by disrupting the integrity of the blood-brain barrier and inhibiting the permeability of the blood-brain barrier^38^,, increasing acetylcholine esterase activity that responsible for degradation acetylcholine neurotransmitter which exerts a reno-protective effect^39,40^, inhibiting cholinergic activity which associated with blocking GABA ergic receptors^41^, making a hyper-stimulatory effect on glutamate receptors that is associated with the loss of Purkinje neurons in the cerebellar cortex, hyper excitatory in cornu Ammonis (CA1) region in the hippocampus that is responsible for memory and learning that ultimately leads to death of pyramidal neurons (major GABAergic neurons) via necrosis^4^.

Brain histological damage was recorded after examination of the cerebellar cortex and CA1 region in the hippocampus by loss of Purkinje and pyramidal neurons in both regions, respectively. As well, our results also proved that, after using a supportive confirmatory tool, morphometry examination in the cerebellar cortex and CA1 region in the hippocampus revealed that several Purkinje and pyramidal neurons, respectively, were increased by a greatly significant degree after PRP injection compared to a positive ischemic group of rats. Based on previous studies that proved brain tissue is adversely affected following renal I/R injury, especially in the CA1 region in the hippocampus, which therefore causes hyper-excitatory in the CA1 region and loss of the number of pyramidal neurons^4^, and loss of the number of Purkinje cells that are known as master GABAergic neurons, it considered experienced results in line with our biochemical findings that refer to diminishing the GABA neurotransmitter level in brain homogenate^41^.

In the present study, non-invasive color-doppler ultrasonography was used to assess renal hemodynamic conditions of the renal microcirculation after 45 min of renal ischemia, as recommended by many authors^42–44^ who declared its role as a prognostic tool in predicting revascularization success after ischemia is relieved. Results of Doppler ultrasonography showed a significant increase in both renal RI and PI (renal vascular resistance indicators) in ischemic rats, which could be attributed to both acute tubular necrosis and interstitial edema within the ischemic solitary kidney, as explained by Değirmenci et al.^45^ who declared that both PI and RI afford an estimate of the degree of interstitial edema within the kidney graft after transplantation.

Darabont et al.^46^ added that renal RI and PI depend mainly on the renal arterial blood flow during the diastolic phase, which is disturbed by increased intrarenal resistance by renal inflammation and intrarenal microvascular damage, the state of I/R injury. Histopathological examination of the 45-minute ischemic kidney in the I/R group confirmed this explanation. Köger et al.^43^ mentioned that high resistive indices (> 0.8) in renal transplant recipients are associated with an increased risk of graft loss and death. Additionally, the elevated RI in I/R rats could be attributed to the impaired creatinine clearance along with elevated creatinine concentrations in such rats, as reported by^47^ who found an inverse correlation between renal resistance index and a reduction in creatinine clearance.

In the same manner, doppler ultrasonography showed a significant decrease in renal artery flow velocity (PSV and TAMV) in 45-minute ischemic kidneys, which are used as early indicators of renal parenchymal resistance and acute kidney injury, as mentioned^48^ who found a moderate negative correlation and a greater than 80% accuracy between a reduction in the magnitude of TAMV and a higher degree of tubular degeneration. Fortunately, the entire treatment group showed significant improvements in all biochemical, histopathological, and creatinine clearance tests, which explain the improvement of all Doppler ultrasonography indices and velocities.

## Conclusion

In summary, we concluded that injection of PRP only or a mixture of PRP and EX. before the reperfusion phase exerts a significantly superior effect on kidney and brain biochemical parameters and enhancement in the regeneration of kidney and brain tissue that nearly comes close to that of normal rats, owing to the distinctive incredible growth factors of PRP. Single-dose injection with exosomes doesn’t exhibit a positive physiological change more than the other two groups. Furthermore, injection before the reperfusion phase exhibited an obvious physiological positive change by enhancing the level of biochemical and histological parameters in both kidney and brain tissue by diminishing the elevation of inflammatory reactions induced by the imbalance between the antioxidant defense enzyme and oxidative stress due to the excessive sudden return of blood flow and re-oxygenation.

## Electronic supplementary material

Below is the link to the electronic supplementary material.

Supplementary Material 1

Supplementary Material 2

## Data Availability

The experimental data and the simulation results that support the findings of this study are available in Mendeley Data repository https://data.mendeley.com/datasets/52z7gz55fg/1 , Abdelsalam, Hani (2024), “Exosome & PRP data”, Mendeley Data, V1, doi: 10.17632/52z7gz55fg.1.

## Abbreviations

BBB: Blood brain barrier
I/R: Ischemia/Reperfusion
AKI: Acute kidney injury
PRP: Platelet rich plasma
Ex.: Exosome
CA: Cornu ammonis
AchE: Acetylcholine esterase
ELISA: Enzyme linked immunosorbent assay
CAT: Catalase
GSH: Glutathione
MDA: Malondialdehyde
GABA: Gamma aminobutyric acid
PCR: Polymerase chain reaction
TNFα: Tumor necrosis factor-alpha
IL6: Interleukin-6
RI: Resistance index
PI: Pulsatility index
PSV: Peak systolic velocity
TAMV: Time average mean velocity
