# Supplementary material for: The ameliorative potential of platelet-rich plasma and exosome on renal ischemia/reperfusion-induced uremic encephalopathy in rats
Source: Sci Rep. 2024 Nov 6;14:26888. doi: 10.1038/s41598-024-77094-2 (PMC11541720; doi:10.1038/s41598-024-77094-2)
Supplement: Supplementary file 1 — Supplementary Material 1 [file 41598_2024_77094_MOESM1_ESM.docx]

**The ameliorative potential of Platelet-rich plasma and exosome on Renal Ischemia/Reperfusion-induced Uremic Encephalopathy in rats**


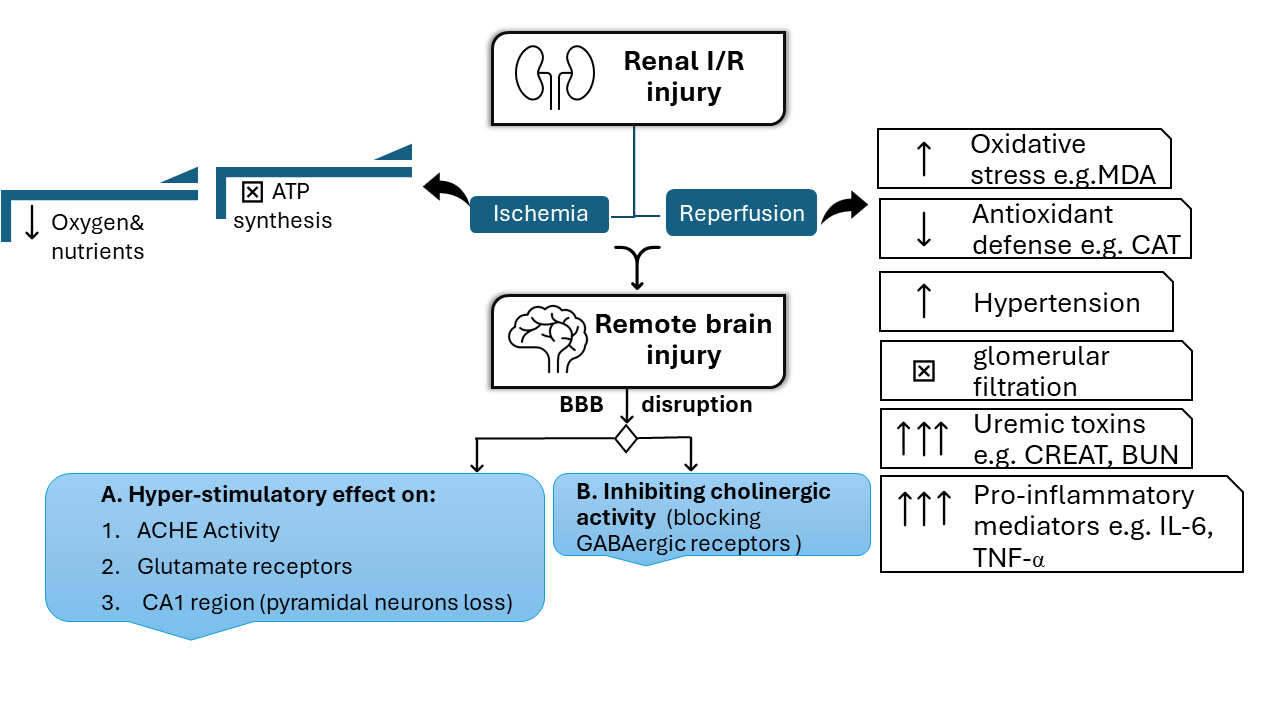


**The graphical abstract illustrates the mechanism of uremia encephalopathy.**
